# Associations Between Child Maltreatment, Inflammation, and Comorbid Metabolic Syndrome to Depressed Mood in a Multiethnic Urban Population: The HELIUS Study

**DOI:** 10.3389/fpsyg.2022.787029

**Published:** 2022-07-14

**Authors:** Fabienne E. M. Willemen, Mirjam van Zuiden, Jasper B. Zantvoord, Susanne R. de Rooij, Bert-Jan H. van den Born, A. Elisabeth Hak, Kathleen Thomaes, Menno Segeren, Leonie K. Elsenburg, Anja Lok

**Affiliations:** ^1^Department of Psychiatry, Amsterdam Neuroscience and Amsterdam Public Health, Amsterdam University Medical Center, University of Amsterdam, Amsterdam, Netherlands; ^2^Department of Child and Adolescent Psychiatry, Amsterdam University Medical Center, Amsterdam, Netherlands; ^3^Department of Epidemiology and Data Science, Amsterdam Public Health Research Institute, Amsterdam University Medical Center, University of Amsterdam, Amsterdam, Netherlands; ^4^Department of Internal and Vascular Medicine, Amsterdam Cardiovascular Sciences, Amsterdam University Medical Center, University of Amsterdam, Amsterdam, Netherlands; ^5^Department of Public Health, Amsterdam University Medical Center, University of Amsterdam, Amsterdam, Netherlands; ^6^Department of Rheumatology and Clinical Immunology, Amsterdam Rheumatology and Immunology Center, Amsterdam University Medical Center, University of Amsterdam, Amsterdam, Netherlands; ^7^Sinai Center, Amstelveen, Netherlands; ^8^Department of Psychiatry, Amsterdam University Medical Center, Vrije University Amsterdam, Amsterdam, Netherlands; ^9^Department of Epidemiology, Health Promotion and Care Innovation, Public Health Service Amsterdam, Amsterdam, Netherlands; ^10^Section of Epidemiology, Department of Public Health, University of Copenhagen, Copenhagen, Denmark; ^11^Department of Public and Occupational Health, Amsterdam University Medical Center, University of Amsterdam, Amsterdam, Netherlands

**Keywords:** child maltreatment, depressed mood, metabolic syndrome, CRP, HELIUS study

## Abstract

**Background:**

Child maltreatment is a common negative experience and has potential long-lasting adverse consequences for mental and physical health, including increased risk for major depressive disorder (MDD) and metabolic syndrome. In addition, child maltreatment may increase the risk for comorbid physical health conditions to psychiatric conditions, with inflammation as an important mediator linking child maltreatment to poor adult health. However, it remains unresolved whether experiencing child maltreatment increases the risk for the development of comorbid metabolic syndrome to MDD. Therefore, we investigated whether child maltreatment increased the risk for comorbid metabolic syndrome to depressed mood. Subsequently, we examined whether C-reactive protein (CRP), as an inflammatory marker, mediated this association. In addition, we investigated whether effects differed between men and women.

**Methods:**

Associations were examined within cross-sectional data from the multiethnic HELIUS study (*N* = 21,617). Adult residents of Amsterdam, Netherlands, self-reported on child maltreatment (distinct and total number of types experienced before the age of 16 years) as well as current depressed mood (PHQ-9 score ≥ 10), and underwent physical examination to assess metabolic syndrome. The CRP levels were assessed in *N* = 5,998 participants. Logistic and linear regressions were applied for binary and continuous outcomes, respectively. All analyses were adjusted for relevant demographic, socioeconomic, and lifestyle characteristics, including ethnicity.

**Results:**

A higher number of maltreatment types as well as distinct types of emotional neglect, emotional abuse, and sexual abuse were significantly associated with a higher risk for current depressed mood. Child maltreatment was not significantly associated with the risk for metabolic syndrome in the whole cohort, nor within individuals with depressed mood. As child maltreatment was not significantly associated with the CRP levels, subsequent mediation analyses were not performed. No significant moderating effects by sex were observed.

**Conclusion:**

In this multiethnic urban cohort, child maltreatment was associated with a higher risk for depressed mood. Contrary to our expectations, child maltreatment was not significantly associated with an increased risk for metabolic syndrome, neither in the whole cohort nor as a comorbid condition in individuals with depressed mood. As the data were cross-sectional and came from a non-clinical adult population, longitudinal perspectives in relation to various stages of the investigated conditions were needed with more comprehensive assessments of inflammatory markers.

## Introduction

During childhood, biopsychological development occurs relatively fast, leading to vulnerability to lifelong adverse health effects of negative influences from within the environment (Hensch, 2005; Fumagalli et al., 2007). Child maltreatment, as reported by 34–62% of healthy adults during their childhood in Western countries, is such a common negative experience that may have potential long-lasting adverse consequences for mental and physical health (Felitti et al., 1998; Gilbert et al., 2015; Hughes et al., 2017; Merrick et al., 2018). Child maltreatment involves both direct (i.e., sexual, physical, emotional abuse, or neglect) and indirect (i.e., parental conflict and parental substance abuse) sources of stress (Felitti et al., 1998; Gilbert et al., 2015; Merrick et al., 2018; World Health Organization [WHO], 2017). Health may be either directly or indirectly influenced by maltreatment, due to abnormal functioning of biological systems leading to disturbances in emotional, cognitive, physical, and social functioning (Pechtel and Pizzagalli, 2011; Danese and McEwen, 2012) or by reinforcement of abnormal behavior (e.g., smoking, physical inactivity, and unhealthy diet), respectively (Anda et al., 1999; Ford et al., 2011; Midei and Matthews, 2011; Al Odhayani et al., 2013; Bellis et al., 2014; Danese and Tan, 2014; Hughes et al., 2017; Elkins et al., 2019).

Child maltreatment has been shown to increase the risk for major depressive disorder (MDD) and metabolic syndrome (Scott et al., 2011; Danese and Tan, 2014; Mandelli et al., 2015; Otte et al., 2016; Hughes et al., 2017). MDD, defined as experiencing a depressive episode lasting at least 2 weeks, is the most prevalent psychiatric disorder affecting 163,000 million people in 2017 worldwide (American Psychiatric Association, 2013; Otte et al., 2016; Gbd 2017 Disease and Injury Incidence and Prevalence Collaborators, 2018). Metabolic syndrome is defined as the presence of a specific set of components that constitute risk factors for cardiovascular disease and type 2 diabetes, including increased abdominal obesity, triglyceride levels, blood pressure, fasting glucose levels, and low high-density lipoprotein (HDL) cholesterol. Metabolic syndrome forms a leading public health concern as it affects approximately one-quarter of the population worldwide in 2018 (Alberti et al., 2005; Gheshlagh et al., 2016; Saklayen, 2018). According to the World Health Organization (WHO), the number of people affected by MDD and metabolic syndrome will increase significantly in the next decades, resulting in an expected higher contribution to overall morbidity and mortality (World Health Organization; Vaccarino et al., 2008; Dzherieva et al., 2011). According to a recent meta-analysis, the prevalence of the co-occurrence of MDD and metabolic syndrome is 30.5% (Vancampfort et al., 2014). Individuals with MDD are at 1.5 times higher odds ratio of developing metabolic syndrome than individuals without MDD (Moradi et al., 2021). Metabolic syndrome thus is a common comorbid condition of MDD (Muhtz et al., 2009; Foley et al., 2010; Pan et al., 2012; Penninx et al., 2013; Whooley and Wong, 2013; Gheshlagh et al., 2016; Hiles et al., 2016; Moradi et al., 2021; Vancampfort et al., 2014). This comorbidity is associated with higher societal costs and a further increase of the burden of disease compared to the isolated occurrence of both disorders (Alberti et al., 2005; Penninx et al., 2013).

Child maltreatment has been found to increase the risk for comorbid physical health conditions to psychiatric conditions, including MDD (Widom et al., 2007; Scott et al., 2011). Three previous studies investigated the association between child maltreatment and comorbid (components of) metabolic syndrome to MDD (McIntyre et al., 2012; Wingenfeld et al., 2017; Hosang et al., 2018). These previous studies showed inconsistent findings regarding the association between child maltreatment and risk for comorbid metabolic syndrome to MDD (McIntyre et al., 2012; Wingenfeld et al., 2017; Hosang et al., 2018). It thus remains unresolved whether experiencing child maltreatment increases the risk for the development of comorbid metabolic syndrome to MDD.

Inflammation may be an important mediator linking child maltreatment to poor adult mental and physical health (Danese et al., 2007, 2009; Widom et al., 2007; Capuron et al., 2008; Berens et al., 2017). Several studies have found that child maltreatment significantly impacts inflammatory markers, including higher C-reactive protein (CRP) (Baumeister et al., 2016). CRP is an acute-phase protein synthesized by the liver. Increased circulating CRP is an indicator of ongoing inflammation and is considered a risk factor for developing metabolic syndrome (Sluzewska et al., 1996; Berk et al., 1997; Hornig et al., 1998; Festa et al., 2000; Han et al., 2002; Coelho et al., 2014). Rethorst et al. (2014) previously demonstrated a positive association between the MDD and CRP levels. In addition, previous studies have shown that CRP, as an inflammatory marker, impacts the association between MDD and metabolic syndrome (Rethorst et al., 2014; Chirinos et al., 2017; Lamers et al., 2020). For instance, a former study has found a positive association between CRP and the presence of comorbid metabolic syndrome within individuals with depressed mood (Rethorst et al., 2014). As a result of the previous research, CRP as an inflammatory marker could be a potential mediator in the assumed association between child maltreatment and the development of comorbid metabolic syndrome to MDD.

Against this background, our primary focus was to investigate whether child maltreatment increased the risk of comorbid occurrence of metabolic syndrome and its individual components to the current depressed mood as a self-reported proxy for MDD. Subsequently, the role of CRP as a mediator for these associations was examined. In all analyses, we investigated potential moderating effects by sex, as an increasing amount of literature shows that associations between child maltreatment and adverse health outcomes can differ between men and women (Garad et al., 2017; Ehrlich et al., 2021). This study used data from the Healthy Life in an Urban Setting (HELIUS) study which is a multiethnic urban cohort including several ethnic groups living in Amsterdam, Netherlands. The design of the HELIUS study forms a representative cross-section of this Western European capital’s population in terms of demographic factors (e.g., socioeconomic status, educational level, and ethnicity) (Stronks et al., 2013; Snijder et al., 2017). With this study, we extend on previous findings within the same population that individual child maltreatment types were associated with a higher risk for current depressed mood, without significant moderating effects of sex (Sunley et al., 2020).

The association between the number of child maltreatment types experienced and the risk for current depressed mood was not yet investigated in this population, nor were associations between child maltreatment, metabolic syndrome, and CRP.

## Materials and Methods

### Participants and Procedure

This study describes baseline data from the HELIUS study which is a multiethnic cohort study performed in Amsterdam, Netherlands. It aims to investigate the impact of ethnicity on common major physical and mental health problems. Between 2011 and 2015, baseline data were collected among *N* = 23,942 Amsterdam residents of South-Asian Surinamese, African Surinamese, Turkish, Moroccan, Ghanaian, and Dutch origin. Full details on the study and recruitment method are described elsewhere (Stronks et al., 2013; Snijder et al., 2017). In brief, individuals between the ages of 18 and 70 years were randomly sampled, stratified by ethnicity, from the municipality register of Amsterdam, and invited to participate. We were able to contact 55% of those invited, either by response card or after a home visit by an ethnically matched interviewer. Of those, 50% agreed to participate (participation rate). Therefore, the overall response rate was 28% with some variations across ethnic groups (33% among Dutch, 31% among Surinamese, 22% among Turks, 21% among Moroccans, and 35% among Ghanaians).

Previous non-response analyses indicated that the HELIUS cohort is representative of the investigated ethnic groups in Amsterdam, as only minor differences in socioeconomic characteristics were observed between participants, non-participants, and non-invited eligible individuals. Written informed consent was obtained prior to any study procedures. Participants completed an extensive questionnaire and a physical examination that included the collection of biological samples. The study was approved by the Institutional Review Board of the Amsterdam University Medical Centers located at Academic Medical Center.

For this study, baseline questionnaire and physical examination data were available for *n* = 22,165 participants. We excluded participants of Javanese Surinamese origin (*n* = 233), other/unknown Surinamese origin (*n* = 267), and participants with unknown ethnic background (*n* = 48) due to their comparatively small sample sizes. This resulted in a total sample size of *n* = 21,617. The CRP levels were determined in random subsamples of *n* = 1,000 participants from each ethnic group (total *n* = 6,000), for whom complete data on cardiovascular measurements and stored blood samples were available. The CRP levels could not be assessed in two participants due to very less material, resulting in *n* = 5,998 participants.

### Measures

#### Child Maltreatment

Participants self-reported on experienced child maltreatment using a short self-report questionnaire derived from the NEMESIS Trauma questionnaire (De Graaf et al., 2010). It contains 4 items, each reflecting a specific type of maltreatment experienced before the age of 16 years, namely, emotional neglect (ignored or unsupported), physical abuse (kicked, hit, bitten, or hurt), emotional abuse (yelled at, insulted, or threatened), and sexual abuse (any unwanted sexual experience). Items were scored on a Likert scale (never-once-sometimes-often-would rather not say). The items inquiring about emotional neglect, physical abuse, and emotional abuse were considered endorsed when experienced sometimes or often. The item inquiring about sexual abuse was considered endorsed when experienced at least once (De Graaf et al., 2010). Subsequently, the total number of endorsed maltreatment types was calculated (range: 0–4). Missing data were imputed (refer to below details on the applied imputation strategy).

#### Depressed Mood

Depressed mood was determined using the self-report questionnaire Patient Health Questionnaire-9 (PHQ-9), which inquiries about the presence of depressive symptoms over the past 2 weeks (Kroenke et al., 2010). Its cross-cultural validity across the ethnic groups in the HELIUS study was previously demonstrated (Galenkamp et al., 2017). The questionnaire consists of nine items, with a Likert response scale ranging from never (0) to nearly every day (3). A maximum of one missing item was allowed, in which case the mean score on the other eight items was used to replace it. The cutoff score ≥ 10 indicated a depressed mood (Kroenke et al., 2001). Both the sensitivity and specificity for current MDD at this cutoff score were previously established to be 0.88 (Kroenke et al., 2001).

#### Metabolic Syndrome

Metabolic syndrome was determined using the harmonized definition of the International Diabetes Federation (Alberti et al., 2009), which considers metabolic syndrome to be present if a minimum of three of five criteria are met: (a) elevated fasting glucose (≥ 5.6 mM, or current use of glucose-lowering medication); (b) elevated blood pressure (systolic ≥ 130 and/or diastolic ≥ 85 mm Hg, or current use of blood pressure-lowering medication); (c) reduced HDL cholesterol (< 1.0 mM for men, < 1.3 mM for women, or current use of lipid-lowering medication); (d) elevated triglycerides (≥ 1.7 mM, or current use of lipid-lowering medication); and (e) elevated waist circumference (ethnic-specific cutoff values; for all women ≥ 80 cm, South-Asian men ≥ 90 cm, and other men ≥ 94 cm).

To assess these criteria, blood samples were collected during the physical examination after overnight fasting. Glucose was determined using spectrophotometry with hexokinase as the primary enzyme, and triglycerides and HDL-C were determined using colorimetric spectrophotometry (Roche Diagnostics, Tokyo, Japan) from heparin plasma. Blood pressure was measured in a seated position using a semiautomatic sphygmomanometer (Microlife WatchBP Home; Microlife AG, Widnau, Switzerland) using appropriate cuff sizes after being seated for at least 5 min. The mean of duplicate measurements was used. Waist circumference was measured during the physical examination, also determined as the mean of duplicate measurements. If the difference between the two measurements was greater than 1.0 cm, a third measurement was obtained and the mean of the two measurements that were closest together was calculated.

#### C-Reactive Protein

Blood samples for CRP determination were collected during the physical examination after overnight fasting. Highly sensitive CRP was determined from heparin plasma using particle-enhanced immunoturbidimetric assay (Cobas 702c analyzer; Roche Diagnostics, Mannheim, Germany).

#### Covariates

Socioeconomic and demographic covariates were age, sex, ethnicity, and educational level. Ethnicity was defined according to the countries of birth of the participant and his/her parents. Participants were considered to be of Dutch origin if the person and both parents were born in Netherlands. Participants were considered to be of non-Dutch origin if the person was either born abroad with at least one parent born abroad (first generation) or was born in Netherlands with both his/her parents born abroad (second generation). Surinamese subgroups (i.e., African and South-Asian) were classified according to self-reported ethnic origin. Dummy variables were created contrasting each ethnic minority group (i.e., South-Asian Surinamese, African Surinamese, Ghanaian, Turkish, and Moroccan) to the Dutch group.

Educational level was used as an indicator of socioeconomic status and was defined as the highest qualification obtained, either in Netherlands or in the country of origin. For educational level, we created dummy variables for lower vocational or general secondary education; intermediate vocational or higher secondary education; and higher vocational education or university, contrasting each to no completed or elementary schooling only as the reference group.

The following information regarding health-behavioral and chronic stress characteristics was also included as they represent potential mediating pathways in the associations under investigation, and we aimed to investigate additional effects of child maltreatment over and above these previously described mediating pathways: smoking (in estimated pack-years), alcohol use in the past 12 months (yes/no), and negative life events experienced in the past 12 months (yes/no), all determined by the self-report questionnaire. In addition, the current use of any of the following psychopharmacological medication was included as covariate: antipsychotics (ATC code N05A), anxiolytics (ATC codes N05BA and N03AE), hypnotics (ATC coded N05C and R06AD02), and antidepressants (ATC coded N06AB, N06AA, N03AF, N03AG, N05AN, and N03AX or without ATC code).

### Statistical Analyses

All analyses were performed using SPSS version 26.0. The total number of endorsed types of maltreatment initially could not be calculated for 8.8% of participants because of one or more missing child maltreatment items (either left at least one of the items blank or answered as “would rather not say,” refer to Supplementary Table 1). Missingness was significantly correlated with all dependent and covariate variables, except smoking. To avoid biased estimations of the investigated associations concerning child maltreatment, missing data for the 4 child maltreatment items were imputed using Markov Chain Monte Carlo imputation with fully conditional specification using predictive mean matching. We included auxiliary variables to account for the fact that missingness was not at random (main effects and two-way interactions among categorical predictors). All variables included in our final analyses were included as auxiliary variables (Alberti et al., 2009). In total, 10 imputations were needed to obtain adequate imputation efficiency. All findings reported concern the pooled results from these 10 imputed datasets (refer to Supplementary Table 1 for a comparison of the child maltreatment prevalence in the pooled imputed vs. non-imputed data). PHQ-9 scores to determine the current depressed mood were missing for *n* = 211 (0.98%), and information on metabolic syndrome diagnosis was missing for *n* = 120 (0.56%); these data were pair-wise deleted from the analyses.

Stepwise logistic regression models were applied to assess the association between the number of experienced maltreatment types and the presence of current depressed mood and metabolic syndrome (diagnosis and its 5 individual components) in the full sample and to assess the association between the number of maltreatment types and the presence of co-morbid metabolic syndrome (diagnosis and its 5 individual components) in participants with current depressed mood only. To aid in the interpretation of observed effects, the number of child maltreatment types was included as an ordinal categorical variable, using indicator contrasts with 0 types experienced as the reference group. These analyses were subsequently repeated including the effects of the four distinct child maltreatment types within one analysis, instead of the total number of types experienced.

For each outcome, the first step included the main effect of the number of experienced maltreatment types, sex, and interaction terms between the number of maltreatment types and sex. The next step included all covariates regarding demographic, socioeconomic, health-behavioral, and chronic stress characteristics and current medication use as described earlier. We refer to these models as the simple and full models, respectively. In the latter model, we assess the associations between child maltreatment and the outcomes of interest over and above these previously described potential confounders and mediators. If the interaction effects between child maltreatment and sex were non-significant, these effects were removed from the respective step of the model and the analysis was repeated including only the main effects of child maltreatment and sex.

The Bonferroni corrections were applied to correct for *K* = 26 regressions performed, *k* = 14 in the full sample (1: depressed mood, 2: metabolic syndrome diagnosis, and 3–7: individual metabolic syndrome components), *k* = 12 in participants with current depressed mood (1: metabolic syndrome diagnosis and 2–6: individual metabolic syndrome components), resulting in α < 0.002.

Subsequently, we investigated potential mediation by CRP. As CRP was not normally distributed, a log10(+1) transformation was applied. We excluded *N* = 27 outliers (0.005%) with standardized log-transformed values > ± 3.29. We first performed a linear regression analysis on the association between the number of maltreatment types endorsed and CRP, followed by a regression including the effects of the four distinct child maltreatment types. For these analyses, we applied the same stepwise approach as described earlier, with an applied Bonferroni correction for *K* = 2 analyses, resulting in α < 0.025. As the association between child maltreatment and CRP proved non-significant, no further analyses regarding this potential mediation effect were performed.

## Results

Sample characteristics of the full sample are shown in Tables 1, 2. On average, participants were 44.3 (*SD* = 13.2) years old. Women constituted 57.8% (*n* = 12,488) of the sample. Within the total sample, the prevalence of current depressed mood was 14.8% (*n* = 3,193). In addition, 31.8% (*n* = 6,482) of the participants within the total sample met the diagnostic criteria for metabolic syndrome. Of the *n* = 3,193 participants with current depressed mood, 38.4% (*n* = 1,221) of the participants also met the criteria for comorbid metabolic syndrome. The average number of experienced maltreatment types was 0.60 (*SD* = 1.06): 31.3% (*n* = 6,174) of participants reported to have experienced at least one child maltreatment type.

**TABLE 1:** Participant characteristics.

	Total sample	Depressed mood		Metabolic syndrome		Depressed Mood with co-morbid metabolic syndrome	
	All (*n* = 21617)	Yes (*n* = 3193)	No (*n* = 18213)	Yes (*n* = 6842)	No (*n* = 14655)	Yes (*n* = 1221)	No (*N* = 1955)
Sex (% female)	12488 (57.8%)	2128 (66.6%)	10233 (56.2%)	3556 (52.0%)	8860 (60.5%)	740 (60.6%)	1377 (70.4%)
Age	44.26 (13.20)	43.35 (12.65)	44.38 (13.31)	51.64 (10.51)	42.47 (13.61)	50.32 (9.91)	39.01 (12.22)
**Ethnicity**							
*Dutch*	4564 (21.1%)	329 (10.3%)	4230 (23.2%)	1077 (15.7%)	3464 (23.6%)	95 (7.8%)	231 (11.8%)
*South-Asian-Surinamese*	3043 (14.1%)	562 (17.6%)	2461 (13.5%)	1388 (20.3%)	1646 (11.2%)	288 (23.6%)	273 (14.0%)
*African-Surinamese*	4151 (19.2%)	444 (13.9%)	3668 (20.1%)	1302 (19.0%)	2815 (19.2%)	171 (14.0%)	270 (13.8%)
*Ghanaian*	2339 (10.8%)	210 (6.6%)	2074 (11.4%)	663 (9.7%)	1661 (11.3%)	65 (5.3%)	144 (7.4%)
*Turkish*	3614 (16.7%)	835 (26.2%)	2731 (15.0%)	1301 (19.0%)	2291 (15.6%)	340 (27.8%)	490 (25.1%)
*Moroccan*	3906 (18.1%)	813 (25.5%)	3049 (16.7%)	1111 (16.2%)	2778 (19.0%)	262 (21.5%)	547 (28.0%)
**Education (highest level completed)**							
*Low*	3818 (17.8%)	835 (26.3%)	2921 (16.1%)	1884 (27.8%)	1916 (13.2%)	444 (36.7%)	388 (20.0%)
*Medium-low*	5633 (26.3%)	901 (28.4%)	4678 (25.9%)	2188 (32.3%)	3411 (23.5%)	388 (32.1%)	508 (26.1%)
*Medium-high*	6235 (29.1%)	944 (29.8%)	5256 (29.1%)	1569 (23.2%)	4623 (31.8%)	267 (22.1%)	670 (34.5%)
*High*	5736 (26.8%)	490 (15.5%)	5234 (28.9%)	1134 (16.7%)	4578 (31.5%)	111 (9.2%)	377 (19.4%)
Any negative life events (past 12 months, yes)	14095 (65.6%)	2707 (84.9%)	11322 (62.2%)	4608 (67.8%)	9410 (64.5%)	1051 (86.1%)	1639 (83.9%)
Alcohol use (past 12 months, yes)	10885 (50.6%)	1200 (37.8%)	9633 (53.1%)	3747 (55.2%)	7785 (53.4%)	375 (30.9%)	819 (42.1%)
Smoking (pack-years)	6.07 (15.18)	7.59 (16.68)	5.81 (14.90)	9.03 (19.46)	4.70 (12.50)	9.81 (18.64)	6.19 (15.14)
**Medication use (current)**							
*Antipsychotics*	221 (1.0%)	98 (3.1%)	122 (0.7%)	110 (1.6%)	110 (0.8%)	57 (4.7%)	41 (2.1%)
*Antidepressants*	810 (3.7%)	374 (11.7%)	423 (2.3%)	360 (5.3%)	445 (3.0%)	167 (13.7%)	206 (10.5%)
*Anxiolytics*	262 (1.2%)	129 (4.0%)	132 (0.7%)	122 (1.8%)	137 (0.9%)	57 (4.7%)	71 (3.6%)
*Hypnotics*	199 (0.9%)	90 (2.8%)	104 (0.6%)	83 (1.2%)	116 (0.8%)	36 (3.0%)	54 (2.8%)

Continuous variables are depicted as mean (SD), categorical variables are depicted as N (%). *Low, No schooling or elementary schooling; Medium-low, Lower vocational or secondary schooling; Medium-high, Intermediate vocational or secondary schooling; High, Higher vocational schooling or university.

**TABLE 2:** Descriptive information regarding child maltreatment, depressed mood, metabolic syndrome, and C-reactive protein (CRP).

	Total sample	Depressed mood		Metabolic syndrome		Depressed mood with co-morbid metabolic syndrome	
	**All** **(*n* = 21617)**	**Yes** **(*n* = 3193)**	**No (*n* = 18213)**	**Yes (*n* = 6842)**	**No (*n* = 14655)**	**Yes** **(*n* = 1221)**	**No** **(*N* = 1955)**
Emotional neglect^$^	4817 (23.6%)	1271 (43.6%)	3534 (20.3%)	1441 (22.6%)	3351 (24.0%)	457 (41.8%)	809 (44.7%)
Emotional abuse^$^	3143 (15.3%)	916 (31.2%)	2216 (12.7%)	951 (14.8%)	2177 (15.6%)	336 (30.2%)	577 (31.9%)
Physical abuse^$^	3145 (15.2%)	797 (26.7%)	2334 (13.3%)	1026 (15.8%)	2106 (15.0%)	299 (26.4%)	497 (27.1%)
Sexual abuse^$^	1704 (8.2%)	412 (13.85)	1289 (7.3%)	485 (7.4%)	1210 (8.6%)	147 (13.0%)	263 (14.2%)
Child maltreatment types, mean number of types experienced^$^	0.60 (1.06)	1.12 (1.34)	0.52 (0.98)	0.58 (1.06)	0.54 (1.01)	1.07 (1.36)	1.15 (1.32)
Child maltreatment types, number of types experienced^$^							
*0 types*	13545 (68.7%)	1348 (49.3%)	12127 (71.8%)	4349 (70.8%)	9124 (67.7%)	539 (52.3%)	801 (47.4%)
*1 type*	2816 (14.3%)	472 (17.3%)	2336 (13.8%)	778 (12.7%)	2021 (15.0%)	167 (16.2%)	303 (17.9%)
*2 types*	1494 (7.6%)	343 (12.6%)	1148 (6.8%)	427 (6.9%)	1061 (7.9%)	111 (10.8%)	231 (13.7%)
*3 types*	1351 (6.9%)	382 (14.0%)	964 (5.7%)	414 (6.7%)	932 (6.9%)	134 (13.0%)	247 (14.6%)
*4 types*	513 (2.6%)	188 (6.9%)	323 (1.9%)	178 (2.9%)	332 (2.5%)	79 (7.7%)	109 (6.4%)
Depressed Mood (past 2 weeks, based on PHQ-9)	3193 (14.8%)	3193 (100%)	0 (0%)	1221 (18.1%)	1955 (13.5%)	1221 (100%)	1955 (100%)
PHQ-9 mean	4.77 (5.21)	14.92 (4.47)	2.99 (2.67)	5.15 (5.73)	4.59 (4.94)	15.35 (4.63)	14.66 (4.35)
Metabolic Syndrome diagnosis	6842 (31.8%)	1221 (38.4%)	5537 (30.6%)	6842 (100%)	0 (0.0%)	1221 (100%)	0 (0.0%)
Metabolic Syndrome component							
*High waist circumference*	14044 (65.0%)	2309 (72.4%)	11579 (63.6%)	6447 (94.3%)	7524 (51.4%)	1164 (95.4%)	1136 (58.1%)
*Raised Triglycerides or lipid lowering medication*	4155 (19.3%)	793 (25.0%)	3314 (18.3%)	3752 (54.9%)	401 (2.7%)	730 (59.8%)	63 (3.2%)
*Reduced HDL cholesterol or lipid lowering medication*	6808 (31.7%)	1326 (41.8%)	5041 (29.8%)	4753 (69.5%)	2047 (14.0%)	945 (77.5%)	380 (19.4%)
*Raised blood pressure or blood pressure lowering medication*	10065 (46.7%)	1420 (44.6%)	8525 (46.9%)	5763 (84.3%)	4249 (29.1%)	970 (79.4%)	443 (22.7%)
*Raised fasting glucose or glucose lowering medication*	6578 (30.6%)	1034 (32.6%)	5466 (30.2%)	4999 (73.2%)	1571 (10.7%)	865 (71.0%)	169 (8.6%)
CRP (mg/L)#	2.59 (4.14)	3.18 (4.16)	2.49 (4.12)	3.39 (4.88)	2.21 (3.67)	3.67 (3.94)	2.87 (4.26)

*Continuous variables are depicted as mean (SD), categorical variables are depicted as N (%). ^$^Child maltreatment descriptive represent the non-imputed data for participants completing the respective items only, descriptive concerning the total number of maltreatment types include only those participants with a fully completed child maltreatment questionnaire (n = 19719). #CRP was available for a random subset of participants (N total sample: 5998; N Depressed mood − yes: 897; N depressed mood − no = 5101; N Metabolic syndrome − yes: 1931; N Metabolic syndrome − no: 4067; N Depressed mood no metabolic syndrome: 552; N depressed mood comorbid metabolic syndrome: 345).*

### Association Between Child Maltreatment and Depressed Mood

Women were at increased risk for depressed mood, but sex did not significantly moderate the association between the number of child maltreatment types and depressed mood (Supplementary Table 2). Therefore, the interaction effects were removed from the models. The number of experienced child maltreatment types was significantly associated with the risk for current depressed mood, both in the simple and full models (Table 3). Similarly, for the four distinct child maltreatment types, sex did not significantly moderate the association with depressed mood (all *p*-Values > 0.002). The experience of emotional neglect [simple: OR: 1.966 (95% CI: 1.763–2.192) *p* < 0.001; full: OR: 1.929 (95% CI: 1.707–2.180) *p* < 0.001] and emotional abuse [simple: OR: 1.621 (95% CI: 1.429–1.838) *p* < 0.001; full: OR: 1.599, 95% CI: 1.400–1.826, *p* < 0.001] was significantly associated with depressed mood in both the simple and full models. The effect of sexual abuse was only significant within the full model [simple: OR: 1.211 (95% CI: 1.069–1.371) *p* = 0.003; full: OR: 1.434, (95% CI 1.246–1.650), *p* < 0.001], while the effect of physical abuse was non-significant in both the simple and full models [simple: OR: 1.192 (95% CI: 1.051–1.354) *p* = 0.007; full: OR: 1.149 (95% CI: 1.007–1.312) *p* = 0.039].

**TABLE 3:** Results of the logistic regression analyses assessing associations between the number of experienced child maltreatment types and the presence of current depressed mood in *N* = 20,740 participants.

	Odds Ratio	*P*	95% Confidence Interval Odds Ratio	
**Simple model:**				
*Child maltreatment – 1 type vs. 0 types*	1.864	<0.001	1.659	2.094
*2 vs. 0*	2.784	<0.001	2.452	3.161
*3 vs. 0*	3.523	<0.001	3.091	4.014
*4 vs. 0*	4.619	<0.001	3.813	5.595
*Sex*− *female vs. male*	1.479	<0.001	1.362	1.606
*Constant*	0.092	<0.001	0.085	0.099
**Full model:**				
*Child maltreatment – 1 type vs. 0 types*	1.869	<0.001	1.641	2.129
*2 vs. 0*	2.819	<0.001	2.452	3.241
*3 vs. 0*	3.376	<0.001	2.919	3.906
*4 vs. 0*	5.156	<0.001	4.162	6.388
*Constant*	0.045	<0.001	0.034	0.060

*Full model: adjusted for sex, age, educational level, smoking, alcohol use, psychopharmacological medications, ethnicity (reference group Dutch), any negative life events (12 months).*

### Association Between Child Maltreatment and Metabolic Syndrome

Men were at increased risk for metabolic syndrome, but sex did not significantly moderate the association between child maltreatment and metabolic syndrome (Supplementary Table 3). The number of experienced maltreatment types was not significantly associated with the risk for metabolic syndrome diagnosis, both in the simple and full models (Table 4).

**TABLE 4:** Results of the logistic regression analysis assessing the association between the number of experienced child maltreatment types and metabolic syndrome diagnosis in *N* = 20,714 participants.

	Odds ratio	*P*	95% confidence interval odds ratio	
**Simple model:**				
*Child maltreatment*− *1 type vs. 0 types*	0.901	0.020	0.826	0.984
*2 vs. 0*	0.937	0.247	0.839	1.046
*3 vs. 0*	0.973	0.649	0.863	1.096
*4 vs. 0*	1.167	0.105	0.968	1.407
*Sex*− *female vs. male*	0.700	<0.001	0.660	0.743
*Constant*	0.577	<0.001	0.550	0.605
**Full model:**				
*Child maltreatment – 1 type vs. 0 types*	0.882	0.013	0.799	0.974
*2 vs. 0*	0.918	0.181	0.811	1.041
*3 vs. 0*	0.822	0.004	0.720	0.938
*4 vs. 0*	1.068	0.527	0.871	1.310
*Constant*	0.014	<0.001	0.011	0.018

*Full model: adjusted for sex, age, educational level, smoking, alcohol use, psychopharmacological medications, ethnicity (reference group Dutch), any negative life events (12 months).*

Similarly, for the four distinct child maltreatment types, sex did not significantly moderate the association with metabolic syndrome, nor were distinct maltreatment types significantly associated with the risk for metabolic syndrome diagnosis, both in the simple and full models (all *p*-Values > 0.002).

The number of experienced maltreatment types (either as main effect or interaction effect with sex) was also not significantly associated with the risk for the presence of the individual components of the metabolic syndrome, except for a significantly decreased risk for the presence of elevated blood pressure upon reporting child maltreatment (refer to Supplementary Tables 4A−E).

Regarding the distinct maltreatment types, the only effect that remained significant within the full model was a significantly decreased risk for the presence of elevated blood pressure upon reporting emotional neglect specifically [simple model: OR: 0.814 (95% CI: 0.747–0.887) *p* < 0.001; full model: OR: 0.807 (95% CI: 0.732–0.889), *p* < 0.001].

The effect of physical abuse was also significant in the simple model, but no longer within the full model [simple: OR: 1.275 (95% CI: 1.160–1.402) *p* < 0.001; full: OR: 1.051 (95% CI: 0.994–1.171) *p* = 0.363]. All other effects were non-significant, including the moderation effects by sex (all *p*-Values > 0.002).

#### Association Between Child Maltreatment and Comorbid Metabolic Syndrome to Depressed Mood

Men were at increased risk for comorbid metabolic syndrome, but sex did not significantly moderate the impact of the number of child maltreatment types on metabolic syndrome (Supplementary Table 5). The number of experienced maltreatment types was not significantly associated with the risk for comorbid metabolic syndrome in participants with current depressed mood, both in the simple and full models (Table 5). The number of experienced maltreatment types was also not significantly associated with the individual components of the metabolic syndrome in participants with current depressed mood, either as main effects or interaction effects with sex (refer to Supplementary Tables 6A−E).

**TABLE 5:** Results of the logistic regression analysis assessing the association between the number of experienced child maltreatment types and co-morbid metabolic syndrome diagnosis to current depressed mood in *N* = 3,061 participants.

	Odds ratio	*P*	95% confidence interval odds ratio	
**Simple model:**				
*Child maltreatment – 1 type vs. 0 types*	0.889	0.270	0.721	1.096
*2 vs. 0*	0.769	0.033	0.604	0.979
*3 vs. 0*	0.815	0.103	0.638	1.042
*4 vs. 0*	1.006	0.971	0.736	1.374
*Sex*− *female vs. male*	0.623	<0.001	0.534	0.727
*Constant*	0.923	0.293	0.795	1.072
**Full model:**				
*Child maltreatment*− *1 type vs. 0 types*	0.957	0.721	0.754	1.215
*2 vs. 0*	0.887	0.397	0.673	1.17
*3 vs. 0*	0.766	0.060	0.58	1.011
*4 vs. 0*	1.136	0.489	0.792	1.63
*Constant*	0.015	<0.001	0.008	0.027

*Full model: adjusted for sex, age, educational level, smoking, alcohol use, psychopharmacological medications, ethnicity (reference group Dutch), any negative life events (12 months).*

Similarly, non-significant results regarding the moderation by sex and main effects on comorbid metabolic syndrome were observed for the four distinct child maltreatment types (all *p*-Values > 0.002).

#### Association Between Child Maltreatment and C-Reactive Protein

Women had significantly higher CRP levels, but sex did not significantly moderate the associations between child maltreatment and CRP (Supplementary Table 7). Therefore, the interaction effects were removed from the models. The number of experienced maltreatment types was not significantly associated with circulating CRP levels in the simple and full models (Table 6). There were also no significant associations between the four distinct maltreatment types and CRP, nor significant moderation effects by sex (all *p*-Values > 0.002) Therefore, mediation analyses for the associations between maltreatment, depressed mood, and (co-morbid) metabolic syndrome were not performed.

**TABLE 6:** Results of the linear regression analysis assessing the association between the number of experienced child maltreatment types and circulating CRP in *N* = 5,879 participants.

	*B*	SE	*t*	*p*
**Simple model:**				
*Child maltreatment – 1 type vs. 0 types*	–0.013	0.011	–1.211	0.226
*2 vs. 0*	0.005	0.015	0.351	0.726
*3 vs. 0*	–0.004	0.016	–0.275	0.783
*4 vs. 0*	0.031	0.024	1.256	0.209
*Sex*− *female vs. male*	0.084	0.007	11.253	<0.001
*Constant*	0.377	0.006	60.443	<0.001
**Full model:**				
*Child maltreatment*− *1 type vs. 0 types*	–0.010	0.010	–0.955	0.340
*2 vs. 0*	0.003	0.015	0.229	0.819
*3 vs. 0*	–0.016	0.015	–1.068	0.286
*4 vs. 0*	0.027	0.024	1.138	0.255
*Constant*	0.215	0.025	8.722	<0.001

*Full model: adjusted for sex, age, educational level, smoking, alcohol use, psychopharmacological medications, ethnicity (reference group Dutch), any negative life events (12 months).*

## Discussion

In this study, we investigated whether child maltreatment increases the risk for the presence of co-morbid metabolic syndrome to the current depressed mood among a representative multiethnic urban cohort from Amsterdam, Netherlands. Extending previous research within the cohort in which we showed that individual types of child maltreatment were associated with a higher risk for current depressed mood (Sunley et al., 2020), we observed that a higher number of experienced child maltreatment types were positively associated with a higher risk for current depressed mood. Contrary to our expectations, child maltreatment was not significantly associated with increased risk for metabolic syndrome, neither in the whole sample nor as a comorbid condition to depressed mood. Subsequently, we aimed to investigate whether CRP as an inflammatory marker mediated these associations. However, this mediation analysis was not performed since the association between child maltreatment and CRP was not significant.

We did not observe significant associations between child maltreatment and increased risk for metabolic syndrome or its separate components within the whole sample, in either men or women. Our findings regarding the absent association between the number of child maltreatment types and metabolic syndrome diagnosis are in line with findings from Li et al. (2019) who neither observed an association between the number of experienced child maltreatment types, as reported both in childhood and adulthood, nor a risk for metabolic syndrome at mid-adulthood in women and men from a birth cohort that included both British-born residents and immigrants. Yet, Li et al. (2019) observed specific associations between distinct types of maltreatment and increased risk for specific components of the metabolic syndrome, with the largest effects observed for physical abuse and childhood neglect (Li et al., 2019), while in our study the distinct child maltreatment types were not associated with increased risk for the metabolic syndrome components. We did, however, unexpectedly observe negative associations between distinct maltreatment types as well as the total number of maltreatment types and the presence of elevated blood pressure. The effects of emotional abuse and having experienced once vs. no types of child maltreatment remained significant in the full model including demographic, socioeconomic, health-behavioral, and chronic stress characteristics and current medication use, and warrant further investigation.

In addition, Midei et al. (2013) also did not observe an association between exposure to physical, sexual, and emotional abuse and risk for metabolic syndrome in premenopausal or early perimenopausal women in midlife. Yet, they did observe that physical abuse specifically increased the risk for incident metabolic syndrome in the following 7 years. This effect was specifically related to two components of metabolic syndrome, namely high waist circumference and high fasting glucose (Midei et al., 2013). Contrastingly, Lee et al. (2014) observed that child maltreatment was associated with increased risk for the presence of a higher number of metabolic syndrome components as well as metabolic syndrome diagnosis in both women and men in mid-adulthood within a US study. Moreover, in this study, emotional and physical abuse increased the risk of developing metabolic syndrome across women and men, whereas sexual abuse only increased the risk for metabolic syndrome in women (Lee et al., 2014). Together these studies suggest that there is no general association between childhood maltreatment and metabolic syndrome, rather associations might differ based on interactions between sex, maltreatment type, participant age, and type of cohort. Furthermore, all these studies including this study were cross-sectional, except for Midei and Matthews (2011) who investigated both cross-sectional and longitudinal data. This hampers the interpretation of the nature of the observed associations. It would, therefore, be of interest to investigate longitudinal associations between the various distinct child maltreatment types and the prospective incidence of metabolic syndrome in the planned follow-up assessments within our cohort, especially since the mean age of our cohort is 44.3 years and the associations between child maltreatment and adverse outcome may strengthen with age (Midei et al., 2013).

Within the participants with current depressed mood, childhood maltreatment was not significantly associated with the risk for the presence of co-morbid metabolic syndrome, nor with its separate components. Again, these effects did not differ between men and women. In concordance with this study, the study by Wingenfeld et al. (2017), Hosang et al. (2018) also did not find an association between self-reported maltreatment and medical comorbidities related to metabolic syndrome (e.g., diabetes type 1 and 2, heart problems, and hypertension) in people with recurrent and current unipolar depression, respectively. However, a cross-sectional study by McIntyre et al. (2012) reported that a history of any self-reported childhood trauma was associated with increased risk for one component of the metabolic syndrome in people with MDD, i.e., lower HDL levels. In our study, we used self-reported current depressed mood in the past 2 weeks as a proxy for probable current MDD, whereas McIntyre et al. (2012), Wingenfeld et al. (2017), Hosang et al. (2018) included a clinical MDD diagnosis. Although it is not completely substitutable for a diagnostic interview, the PHQ-9 has been repeatedly found to be a valid diagnostic screener to measure current depressed mood and has good sensitivity and specificity for the detection of current MDD (Kroenke et al., 2001). Nonetheless, potentially our use of a more lenient definition of depressed mood might explain the contrasting findings between studies, and this should be further investigated in the future.

In support of our approach to investigating comorbid metabolic syndrome to depressed mood, a meta-analysis by Blaine (2008) showed that people with depression were more likely to develop obesity over time than people without depression. Conversely, in a 5-year-longitudinal study, Roberts et al. (2003) showed that the presence of obesity at baseline predicted the subsequent development of a major depressive episode among middle-aged men and women. A recent meta-analysis extends this evidence, as they observed that three surrogate measures of insulin resistance at baseline and development of prediabetes within the first 2 years were associated with a greater risk of incident MDD among healthy adults with no history of MDD or anxiety in a 9-year follow-up (Watson et al., 2021). These findings emphasize the need to also investigate the association between child maltreatment and comorbid depressed mood to metabolic syndrome, preferably in a design that allows for drawing temporal causal inferences. Again, longitudinal studies could help to determine the direction of the effect in the relationship between depression and metabolic syndrome and potential effects of child maltreatment.

We also aimed to investigate whether circulating CRP levels mediated the association between child maltreatment and comorbid metabolic syndrome in participants with current depressed mood. The actual mediation analysis was not performed since the main association between child maltreatment and CRP levels was not significant. Our finding of the absence of an association between childhood maltreatment and CRP is in line with a recent systematic review (Kerr et al., 2021) that showed that previous retrospective studies with retrospective reporting on child maltreatment in adulthood including non-clinical samples have also found no association between maltreatment and CRP. Yet, in contrast, three prospective studies using objective assessment of maltreatment already in childhood, including non-clinical samples, all observed that maltreatment was associated with elevated CRP levels (Danese et al., 2007; Nikulina and Widom, 2014; Osborn and Widom, 2020).

Baldwin et al. (2019) previously investigated the validity of self-report vs. objective measurements of maltreatment. They emphasize that retrospective self-report measures should be used with caution since it may not accurately reflect the experiences of maltreatment due to memory biases which can result in overreporting or underreporting of actual experiences (Baldwin et al., 2019). This might be especially relevant for individuals with depressed mood or MDD, as this might influence the recall and interpretation of previous negative events such as child maltreatment (negative cognitive bias) (Łosiak et al., 2019). Therefore, future longitudinal studies should ideally use a combination of retrospective self-report and prospective objective measures to capture the experiences of maltreatment.

xsx Furthermore, we measured inflammation with just one single marker, i.e., CRP. Although CRP is an established indicator of ongoing inflammation and is commonly used as a diagnostic biomarker for inflammation, individual inflammatory markers in general do not adequately reflect all aspects of inflammatory processes (Del Giudice and Gangestad, 2018). Therefore, future studies should ideally include a more comprehensive assessment of inflammatory markers for a more detailed investigation of the potential mediating role of inflammatory processes.

Moreover, in our multiethnic cohort, the minority groups were previously found to have an increased risk for multiple diseases, including cardiovascular diseases and asthma (Aarab et al., 2019; Perini et al., 2019). Therefore, the observed elevated CRP levels in four out of the five minority groups compared to the ethnic Dutch reference group are not unexpected. Yet, it is well conceivable that these larger effects confounded more subtle associations between child maltreatment and CRP, even though we accounted for ethnicity in our analyses.

### Strengths and Limitations

This study investigated child maltreatment and comorbid metabolic syndrome to current depressed mood in a large representative urban multiethnic cohort in Western Europe (Snijder et al., 2017). The assumed generalizability of the findings is further supported by the fact that the self-reported prevalence rate of child maltreatment of approximately 30% is similar to that previously reported in the general Dutch population (De Graaf et al., 2010).

Nevertheless, our study has several limitations that should be addressed. First, as already mentioned earlier as this study had a cross-sectional design, it is not possible to draw causal or inferences on directionality of effects. Our study suggests that child maltreatment is not associated with increased risk for comorbid metabolic syndrome to depressed mood. However, there is a possibility that the effects of child maltreatment do exist at a more nuanced level and will only come to light when depressed mood and metabolic syndrome development are investigated longitudinally. Next, this study used self-report questionnaires which could have led to decreased reporting reliability. However, in contrast, self-report questionnaires may also represent the respondent’s perspective more reliably and stimulate disclosing as compared to interview situations, specifically on sensitive topics, i.e., child maltreatment and depressed mood (McNeeley, 2012). In addition, with respect to child maltreatment, recall bias may have occurred, as child maltreatment may happen at a young age in which children do not have the cognitive ability to recall these events. Moreover, our measure of child maltreatment only included four types of maltreatment (i.e., emotional neglect, psychological abuse, physical abuse, and sexual abuse), which obviously does not cover all the various types of child maltreatment. The measurement of child maltreatment used was developed for use in a Dutch community population. Unfortunately, the questionnaire’s cross-cultural validity has not yet been investigated, and we cannot exclude differences in the reliability of retrospective child maltreatment reporting between the different ethnic groups included in our study population, nor in the interpretation of what constitutes child maltreatment and was, therefore, endorsed as such in the questionnaire. Furthermore, in line with previous investigations on the associations between child maltreatment and adverse physical health outcomes within the HELIUS study (Bakema et al., 2020), we solely investigated the associations between the occurrence of the distinct child maltreatment types and the total number of experienced maltreatment types. The developmental timing and chronicity of maltreatment was also not assessed, which could have had an impact on the results, as previous studies have shown that the exact timing of maltreatment within childhood impacts the occurrence of physical and mental health and neurobiological correlates in adulthood (Pechtel et al., 2014).

Finally, while we included ethnicity as a covariate in our analyses, we did not explicitly investigate whether there were differences in the associations between child maltreatment and the various outcomes of interest between our included ethnic groups, nor in the associations of these outcomes with the included covariates on demographic, socioeconomic, health-behavioral, and chronic stress characteristics and current medication use in the statistical models.

## Conclusion and Future Implications

This multiethnic urban cohort study provides insights into the association between child maltreatment and comorbid metabolic syndrome in people with current depressed mood, and the potential mediating effect of the inflammatory marker CRP. No significant association was found between child maltreatment and comorbid metabolic syndrome in people with current depressed mood. As child maltreatment was not significantly associated with circulating CRP, mediation was excluded. The observed associations did not differ between men and women. Although our cohort was cross-sectional and consisted of a non-clinical adult population, our study findings provide additional support for the notion that although child maltreatment has debilitating effects on wellbeing and many health outcomes, it does not unequivocally increase the risk for all adverse health outcomes at any moment throughout life. In future research, it is essential to gain prospective longitudinal perspectives regarding different stages of the examined conditions with more comprehensive measures of maltreatment, depressed mood, metabolic syndrome, and inflammation.

## Data Availability Statement

The data that support the findings of this study are available upon request from the HELIUS research cohort, but restrictions apply to the availability of these data which were used under license for the current study, and so are not publicly available. Galenkamp is the Scientific Coordinator of HELIUS and may be contacted with further questions (h.galenkamp@amsterdamumc.nl).

## Ethics Statement

The studies involving human participants were reviewed and approved by the Institutional Review Board of the Amsterdam University Medical Centers, location Academic Medical Center. The patients/participants provided their written informed consent to participate in this study.

## Author Contributions

MZ and AL conceptualized and designed the current study. FW, SR, B-JB, AH, KT, MS, and LE contributed to the conceptualization and interpretation of results. MZ organized the database and performed the statistical analyses. FW and MZ wrote the first draft of the manuscript. AL and JZ wrote sections of the manuscript. All authors contributed to manuscript revision, read and approved the submitted version.

## Conflict of Interest

The authors declare that the research was conducted in the absence of any commercial or financial relationships that could be construed as a potential conflict of interest.

## Publisher’s Note

All claims expressed in this article are solely those of the authors and do not necessarily represent those of their affiliated organizations, or those of the publisher, the editors and the reviewers. Any product that may be evaluated in this article, or claim that may be made by its manufacturer, is not guaranteed or endorsed by the publisher.
