# Supplementary material for: Associations Between Child Maltreatment, Inflammation, and Comorbid Metabolic Syndrome to Depressed Mood in a Multiethnic Urban Population: The HELIUS Study
Source: Front Psychol. 2022 Jul 14;13:787029. doi: 10.3389/fpsyg.2022.787029 (PMC9331167; doi:10.3389/fpsyg.2022.787029)
Supplement: Supplementary file 1 [file Data_Sheet_1.docx]

***Supplementary tables for ‘Associations between child maltreatment. inflammation and comorbid metabolic syndrome to depressed mood in a multi-ethnic urban population: The HELIUS study’***

**Supplementary Table 1. Comparison of the prevalence of the self-reported child maltreatment types in the raw. non-imputed data (n=19719 with complete data) compared to the pooled imputed data (n=21617 with complete data) within the whole sample.**

|  | Non-imputed data | Imputed data |
| --- | --- | --- |
| Sexual abuse |  |  |
| *Never* | 19049 (88.1%) | 19411 (89.8%) |
| *Once* | 874 (4.0%) | 1281 (5.9%) |
| *Sometimes* | 575 (2.7%) | 666 (3.1%) |
| *Regularly* | 255 (1.2%) | 259 (1.2%) |
| *Would rather not say* | 583 (2.7%) | na |
| *Blank* | 281 (1.3%) | na |
| Emotional neglect |  |  |
| *Never* | 14930 (69.1%) | 15255 (70.6%) |
| *Once* | 689 (3.2%) | 1128 (5.2%) |
| *Sometimes* | 2868 (13.3%) | 3183 (14.7%) |
| *Regularly* | 1949 (9.0%) | 2051 (9.5%) |
| *Would rather not say* | 879 (4.1%) | na |
| *Blank* | 302 (1.4%) | na |
| Emotional abuse |  |  |
| *Never* | 16913 (78.2%) | 17278 (79.9%) |
| *Once* | 483 (2.2%) | 921 (4.3%) |
| *Sometimes* | 1947 (9.0%) | 2175 (10.1%) |
| *Regularly* | 1196 (5.5%) | 1243 (5.8%) |
| *Would rather not say* | 732 (3.4%) | na |
| *Blank* | 346 (1.6%) | na |
| Physical Abuse |  |  |
| *Never* | 16653 (77.0%) | 16957 (78.4%) |
| *Once* | 892 (4.1%) | 1284 (5.9%) |
| *Sometimes* | 2319 (10.7%) | 2514 (11.6%) |
| *Regularly* | 826 (4.0%) | 862 (4.0%) |
| *Would rather not say* | 598 (2.8%) | na |
| *Blank* | 329 (1.5%) | na |
| Child maltreatment. mean number of types endorsed | 0.60 (1.06) | 0.66 (1.07) |
| Child maltreatment types. number of types endorsed |  |  |
| *0* | 13545 (68.7%) | 14096 (65.2%) |
| *1* | 2816 (14.3%) | 3498 (16.2%) |
| *2* | 1494 (7.6%) | 1904 (8.8%) |
| *3* | 1351 (6.9%) | 1548 (7.2%) |
| *4* | 513 (2.6%) | 571 (2.6%) |

**Supplementary Table 2. Results of the simple model assessing associations between number of experienced child maltreatment types and presence of current depressed mood including maltreatment x sex interaction effects in N=20740 participants.**

|  | Odds Ratio | P | 95% Confidence Interval  Odds Ratio | |
| --- | --- | --- | --- | --- |
| *Child maltreatment - 1 type vs 0 types* | 2.257 | <.001 | 1.862 | 2.736 |
| *2 vs 0* | 3.463 | <.001 | 2.797 | 4.288 |
| *3 vs 0* | 4.401 | <.001 | 3.560 | 5.440 |
| *4 vs 0* | 7.543 | <.001 | 5.131 | 11.089 |
| *Sex - female vs male* | 1.754 | <.001 | 1.557 | 1.976 |
| *Sex * Child maltreatment – 1 type vs 0 types* | 0.745 | .018 | 0.583 | 0.951 |
| *Sex * Child maltreatment - 2 types vs 0 types* | 0.716 | .012 | 0.553 | 0.928 |
| *Sex* Child maltreatment - 3 types vs 0 types* | 0.705 | .009 | 0.542 | 0.917 |
| *Sex * Child maltreatment - 4 types vs 0 types* | 0.521 | .004 | 0.335 | 0.810 |
| *Constant* | 0.082 | <.001 | 0.075 | 0.090 |

**Supplementary Table 3. Results of the simple model assessing associations between number of experienced child maltreatment types and metabolic syndrome diagnosis including maltreatment x sex interaction effects in N=20714 participants.**

|  | Odds Ratio | P | 95% Confidence Interval  Odds Ratio | |
| --- | --- | --- | --- | --- |
| *Child maltreatment - 1 type vs 0 types* | 0.943 | .388 | 0.826 | 1.077 |
| *2 vs 0* | 1.026 | .770 | 0.861 | 1.223 |
| *3 vs 0* | 0.975 | .779 | 0.814 | 1.167 |
| *4 vs 0* | 1.116 | .580 | 0.755 | 1.651 |
| *Sex - female vs male* | 0.718 | <.001 | 0.668 | 0.773 |
| *Sex * Child maltreatment – 1 type vs 0 types* | 0.921 | .361 | 0.771 | 1.099 |
| *Sex * Child maltreatment - 2 types vs 0 types* | 0.852 | .174 | 0.677 | 1.073 |
| *Sex* Child maltreatment - 3 types vs 0 types* | 0.995 | .969 | 0.784 | 1.263 |
| *Sex * Child maltreatment - 4 types vs 0 types* | 1.051 | .827 | 0.670 | 1.650 |
| *Constant* | 0.569 | <.001 | 0.539 | 0.600 |

**Supplementary Table 4A. Results of the logistic regression analysis assessing the association between number of experienced child maltreatment types and metabolic syndrome component elevated fasting glucose (≥ 5.6 mM. or current use of glucose-lowering medication) in N=20719 participants.**

|  | Odds Ratio | P | 95% Confidence Interval  Odds Ratio | |
| --- | --- | --- | --- | --- |
| Simple model with interactions: |  |  |  |  |
| *Child maltreatment - 1 type vs 0 types* | 0.993 | .915 | 0.876 | 1.126 |
| *2 vs 0* | 0.984 | .852 | 0.835 | 1.161 |
| *3 vs 0* | 0.939 | .485 | 0.787 | 1.120 |
| *4 vs 0* | 1.438 | .059 | 0.987 | 2.097 |
| *Sex - female vs male* | 0.501 | <.001 | 0.466 | 0.540 |
| *Sex * Child maltreatment – 1 type vs 0 types* | 0.868 | .109 | 0.730 | 1.032 |
| *Sex * Child maltreatment - 2 types vs 0 types* | 0.866 | .222 | 0.687 | 1.091 |
| *Sex* Child maltreatment - 3 types vs 0 types* | 1.038 | .764 | 0.815 | 1.321 |
| *Sex * Child maltreatment - 4 types vs 0 types* | 0.814 | .360 | 0.524 | 1.266 |
| *Constant* | 0.651 | <.001 | 0.618 | 0.686 |
| Simple model without interactions: |  |  |  |  |
| *Child maltreatment - 1 type vs 0 types* | 0.924 | .082 | 0.845 | 1.010 |
| *2 vs 0* | 0.912 | .120 | 0.812 | 1.024 |
| *3 vs 0* | 0.958 | .498 | 0.848 | 1.084 |
| *4 vs 0* | 1.246 | .024 | 1.030 | 1.508 |
| *Sex - female vs male* | 0.484 | <.001 | 0.456 | 0.514 |
| *Constant* | 0.663 | <.001 | 0.632 | 0.695 |
| Full model without: |  |  |  |  |
| *Child maltreatment - 1 type vs 0 types* | 0.922 | .125 | 0.831 | 1.023 |
| *2 vs 0* | 0.892 | .084 | 0.783 | 1.016 |
| *3 vs 0* | 0.827 | .006 | 0.724 | 0.946 |
| *4 vs 0* | 1.162 | .154 | 0.945 | 1.428 |
| *Constant* | 0.022 | <.001 | 0.018 | 0.028 |

Full model: adjusted for sex, age, educational level, smoking, alcohol use, psychopharmacological medications, ethnicity (reference group Dutch), any negative life events (12 months).

**Supplementary Table 4B. Results of the logistic regression analysis assessing the association between number of experienced child maltreatment types and metabolic syndrome component elevated blood pressure (systolic ≥ 130 and/ or diastolic ≥ 85 mm Hg. or current use of blood pressure–lowering medication) in N=20786 participants.**

|  | Odds Ratio | P | 95% Confidence Interval  Odds Ratio | |
| --- | --- | --- | --- | --- |
| Simple model with interactions: |  |  |  |  |
| *Child maltreatment - 1 type vs 0 types* | 0.809 | .001 | 0.713 | 0.918 |
| *2 vs 0* | 1.007 | .939 | 0.850 | 1.192 |
| *3 vs 0* | 0.922 | .354 | 0.778 | 1.094 |
| *4 vs 0* | 0.812 | .287 | 0.552 | 1.193 |
| *Sex - female vs male* | 0.517 | <.001 | 0.482 | 0.554 |
| *Sex * Child maltreatment – 1 type vs 0 types* | 1.159 | .085 | 0.980 | 1.371 |
| *Sex * Child maltreatment - 2 types vs 0 types* | 0.888 | .274 | 0.718 | 1.098 |
| *Sex* Child maltreatment - 3 types vs 0 types* | 1.163 | .193 | 0.927 | 1.459 |
| *Sex * Child maltreatment - 4 types vs 0 types* | 1.459 | .088 | 0.945 | 2.252 |
| *Constant* | 1.296 | <.001 | 1.231 | 1.365 |
| Simple model without interactions: |  |  |  |  |
| *Child maltreatment - 1 type vs 0 types* | 0.881 | .002 | 0.814 | 0.954 |
| *2 vs 0* | 0.935 | .221 | 0.840 | 1.041 |
| *3 vs 0* | 1.006 | .912 | 0.899 | 1.126 |
| *4 vs 0* | 1.081 | .392 | 0.904 | 1.292 |
| *Sex - female vs male* | 0.533 | <.001 | 0.504 | 0.564 |
| *Constant* | 1.274 | <.001 | 1.216 | 1.334 |
| Full model without: |  |  |  |  |
| *Child maltreatment - 1 type vs 0 types* | 0.833 | <.001 | 0.760 | 0.913 |
| *2 vs 0* | 0.884 | .048 | 0.782 | 0.999 |
| *3 vs 0* | 0.819 | .003 | 0.719 | 0.933 |
| *4 vs 0* | 0.823 | .060 | 0.672 | 1.008 |
| *Constant* | 0.045 | <.001 | 0.037 | 0.056 |

Full model: adjusted for sex, age, educational level, smoking, alcohol use, psychopharmacological medications, ethnicity (reference group Dutch), any negative life events (12 months).

**Supplementary Table 4C. Results of the logistic regression analysis assessing the association between number of experienced child maltreatment types and metabolic syndrome component reduced HDL cholesterol (< 1.0 mM for men. < 1.3 mM for women. or current use of lipid-lowering medication) in N=20725 participants.**

|  | Odds Ratio | P | 95% Confidence Interval  Odds Ratio | |
| --- | --- | --- | --- | --- |
| Simple model with interactions: |  |  |  |  |
| *Child maltreatment - 1 type vs 0 types* | 0.955 | .520 | 0.829 | 1.100 |
| *2 vs 0* | 1.018 | .847 | 0.848 | 1.222 |
| *3 vs 0* | 1.047 | .641 | 0.864 | 1.269 |
| *4 vs 0* | 1.015 | .944 | 0.676 | 1.522 |
| *Sex - female vs male* | 1.171 | <.001 | 1.088 | 1.261 |
| *Sex * Child maltreatment – 1 type vs 0 types* | 1.003 | .974 | 0.834 | 1.206 |
| *Sex * Child maltreatment - 2 types vs 0 types* | 0.889 | .338 | 0.699 | 1.131 |
| *Sex* Child maltreatment - 3 types vs 0 types* | 0.954 | .706 | 0.745 | 1.221 |
| *Sex * Child maltreatment - 4 types vs 0 types* | 0.957 | .851 | 0.605 | 1.514 |
| *Constant* | 0.423 | <.001 | 0.400 | 0.447 |
| Simple model without interactions: |  |  |  |  |
| *Child maltreatment - 1 type vs 0 types* | 0.957 | .336 | 0.875 | 1.047 |
| *2 vs 0* | 0.947 | .336 | 0.847 | 1.058 |
| *3 vs 0* | 1.018 | .775 | 0.902 | 1.149 |
| *4 vs 0* | 0.983 | .860 | 0.812 | 1.190 |
| *Sex - female vs male* | 1.155 | <.001 | 1.088 | 1.227 |
| *Constant* | 0.426 | <.001 | 0.405 | 0.448 |
| Full model without: |  |  |  |  |
| *Child maltreatment - 1 type vs 0 types* | 0.984 | .739 | 0.898 | 1.080 |
| *2 vs 0* | 0.968 | .587 | 0.860 | 1.089 |
| *3 vs 0* | 0.948 | .407 | 0.835 | 1.076 |
| *4 vs 0* | 0.990 | .921 | 0.811 | 1.209 |
| *Constant* | 0.099 | <.001 | 0.081 | 0.122 |

Full model: adjusted for sex, age, educational level, smoking, alcohol use, psychopharmacological medications, ethnicity (reference group Dutch), any negative life events (12 months).

**Supplementary Table 4D. Results of the logistic regression analysis assessing the association between number of experienced child maltreatment types and metabolic syndrome component elevated triglycerides (≥ 1.7 mM. or current use of lipid-lowering medication) in N=20724 participants.**

|  | Odds Ratio | P | 95% Confidence Interval  Odds Ratio | |
| --- | --- | --- | --- | --- |
| Simple model with interactions: |  |  |  |  |
| *Child maltreatment - 1 type vs 0 types* | 1.005 | .940 | 0.875 | 1.156 |
| *2 vs 0* | 1.024 | .816 | 0.841 | 1.247 |
| *3 vs 0* | 1.182 | .091 | 0.974 | 1.435 |
| *4 vs 0* | 1.054 | .810 | 0.686 | 1.620 |
| *Sex - female vs male* | 0.464 | <.001 | 0.424 | 0.507 |
| *Sex * Child maltreatment – 1 type vs 0 types* | 1.011 | .921 | 0.818 | 1.249 |
| *Sex * Child maltreatment - 2 types vs 0 types* | 1.012 | .934 | 0.764 | 1.340 |
| *Sex* Child maltreatment - 3 types vs 0 types* | 0.936 | .643 | 0.709 | 1.237 |
| *Sex * Child maltreatment - 4 types vs 0 types* | 1.197 | .478 | 0.728 | 1.968 |
| *Constant* | 0.346 | <.001 | 0.326 | 0.367 |
| Simple model without interactions: |  |  |  |  |
| *Child maltreatment - 1 type vs 0 types* | 1.011 | .845 | 0.909 | 1.124 |
| *2 vs 0* | 1.030 | .667 | 0.900 | 1.178 |
| *3 vs 0* | 1.146 | .063 | 0.992 | 1.323 |
| *4 vs 0* | 1.197 | .133 | 0.947 | 1.512 |
| *Sex - female vs male* | 0.465 | <.001 | 0.433 | 0.498 |
| *Constant* | 0.346 | <.001 | 0.328 | 0.365 |
| Full model without: |  |  |  |  |
| *Child maltreatment - 1 type vs 0 types* | 0.959 | .471 | 0.857 | 1.074 |
| *2 vs 0* | 0.973 | .708 | 0.842 | 1.124 |
| *3 vs 0* | 0.976 | .756 | 0.835 | 1.140 |
| *4 vs 0* | 1.115 | .403 | 0.864 | 1.438 |
| *Constant* | 0.013 | <.001 | 0.010 | 0.017 |

Full model: adjusted for sex, age, educational level, smoking, alcohol use, psychopharmacological medications, ethnicity (reference group Dutch), any negative life events (12 months).

**Supplementary Table 4E. Results of the logistic regression analysis assessing the association between number of experienced child maltreatment types and metabolic syndrome component elevated waist circumference (ethnic specific cut-off values; for all women ≥ 80 cm. South-Asian men ≥ 90 cm and other men ≥ 94 cm) in N=20812 participants.**

|  | Odds Ratio | P | 95% Confidence Interval  Odds Ratio | |
| --- | --- | --- | --- | --- |
| Simple model with interactions: |  |  |  |  |
| *Child maltreatment - 1 type vs 0 types* | 0.968 | .610 | 0.855 | 1.096 |
| *2 vs 0* | 1.147 | .106 | 0.971 | 1.356 |
| *3 vs 0* | 1.052 | .572 | 0.883 | 1.253 |
| *4 vs 0* | 1.279 | .215 | 0.866 | 1.890 |
| *Sex - female vs male* | 3.097 | <.001 | 2.879 | 3.333 |
| *Sex * Child maltreatment – 1 type vs 0 types* | 0.916 | .301 | 0.775 | 1.082 |
| *Sex * Child maltreatment - 2 types vs 0 types* | 0.773 | .022 | 0.621 | 0.963 |
| *Sex* Child maltreatment - 3 types vs 0 types* | 1.059 | .653 | 0.824 | 1.363 |
| *Sex * Child maltreatment - 4 types vs 0 types* | 0.916 | .713 | 0.576 | 1.459 |
| *Constant* | 1.007 | .792 | 0.957 | 1.060 |
| Simple model without interactions: |  |  |  |  |
| *Child maltreatment - 1 type vs 0 types* | 0.925 | .076 | 0.849 | 1.008 |
| *2 vs 0* | 0.997 | .964 | 0.893 | 1.114 |
| *3 vs 0* | 1.083 | .189 | 0.961 | 1.220 |
| *4 vs 0* | 1.212 | .068 | 0.986 | 1.490 |
| *Sex - female vs male* | 2.994 | <.001 | 2.822 | 3.176 |
| *Constant* | 1.024 | .322 | 0.977 | 1.072 |
| Full model without: |  |  |  |  |
| *Child maltreatment - 1 type vs 0 types* | 0.916 | .065 | 0.834 | 1.006 |
| *2 vs 0* | 0.971 | .640 | 0.858 | 1.098 |
| *3 vs 0* | 0.932 | .295 | 0.818 | 1.063 |
| *4 vs 0* | 1.057 | .617 | 0.850 | 1.315 |
| *Constant* | 0.053 | <.001 | 0.043 | 0.065 |

Full model: adjusted for sex, age, educational level, smoking, alcohol use, psychopharmacological medications, ethnicity (reference group Dutch), any negative life events (12 months).

**Supplementary Table 5. Results of the simple model assessing associations between number of experienced child maltreatment types and co-morbid metabolic syndrome diagnosis to current depressed mood including maltreatment x sex interaction effects in N=3061 participants.**

|  | Odds Ratio | P | 95% Confidence Interval  Odds Ratio | |
| --- | --- | --- | --- | --- |
| *Child maltreatment - 1 type vs 0 types* | 0.925 | .649 | 0.661 | 1.294 |
| *2 vs 0* | 0.775 | .224 | 0.514 | 1.170 |
| *3 vs 0* | 0.744 | .132 | 0.506 | 1.093 |
| *4 vs 0* | 0.895 | .728 | 0.477 | 1.677 |
| *Sex - female vs male* | 0.614 | <.001 | 0.488 | 0.773 |
| *Sex * Child maltreatment – 1 type vs 0 types* | 0.937 | .768 | 0.609 | 1.443 |
| *Sex * Child maltreatment - 2 types vs 0 types* | 0.985 | .955 | 0.581 | 1.67 |
| *Sex* Child maltreatment - 3 types vs 0 types* | 1.163 | .539 | 0.719 | 1.882 |
| *Sex * Child maltreatment - 4 types vs 0 types* | 1.169 | .673 | 0.566 | 2.415 |
| *Constant* | 0.932 | .459 | 0.773 | 1.123 |

**Supplementary Table 6A. Results of the logistic regression analysis assessing the association between number of experienced child maltreatment types and metabolic syndrome component elevated fasting glucose (≥ 5.6 mM. or current use of glucose-lowering medication) in N=3060 participants with depressed mood.**

|  | Odds Ratio | P | 95% Confidence Interval  Odds Ratio | |
| --- | --- | --- | --- | --- |
| Simple model with interactions: |  |  |  |  |
| *Child maltreatment - 1 type vs 0 types* | 1.143 | .442 | 0.813 | 1.607 |
| *2 vs 0* | 0.913 | .649 | 0.617 | 1.352 |
| *3 vs 0* | 0.956 | .815 | 0.658 | 1.390 |
| *4 vs 0* | 1.011 | .974 | 0.532 | 1.919 |
| *Sex - female vs male* | 0.548 | <.001 | 0.433 | 0.692 |
| *Sex * Child maltreatment – 1 type vs 0 types* | 0.730 | .155 | 0.473 | 1.126 |
| *Sex * Child maltreatment - 2 types vs 0 types* | 0.807 | .419 | 0.478 | 1.360 |
| *Sex* Child maltreatment - 3 types vs 0 types* | 0.854 | .523 | 0.526 | 1.387 |
| *Sex * Child maltreatment - 4 types vs 0 types* | 0.854 | .681 | 0.404 | 1.809 |
| *Constant* | 0.754 | .003 | 0.625 | 0.909 |
| Simple model without interactions: |  |  |  |  |
| *Child maltreatment - 1 type vs 0 types* | 0.943 | .605 | 0.756 | 1.177 |
| *2 vs 0* | 0.800 | .081 | 0.622 | 1.028 |
| *3 vs 0* | 0.867 | .259 | 0.676 | 1.112 |
| *4 vs 0* | 0.912 | .590 | 0.651 | 1.276 |
| *Sex - female vs male* | 0.484 | <.001 | 0.413 | 0.567 |
| *Constant* | 0.815 | .008 | 0.701 | 0.949 |
| Full model without: |  |  |  |  |
| *Child maltreatment - 1 type vs 0 types* | 1.088 | .521 | 0.840 | 1.408 |
| *2 vs 0* | 0.945 | .701 | 0.709 | 1.260 |
| *3 vs 0* | 0.827 | .180 | 0.627 | 1.091 |
| *4 vs 0* | 0.965 | .854 | 0.658 | 1.414 |
| *Constant* | 0.007 | <.001 | 0.004 | 0.014 |

Full model: adjusted for sex, age, educational level, smoking, alcohol use, psychopharmacological medications, ethnicity (reference group Dutch), any negative life events (12 months).

**Supplementary Table 6B. Results of the logistic regression analysis assessing the association between number of experienced child maltreatment types and metabolic syndrome component elevated blood pressure (systolic ≥ 130 and/ or diastolic ≥ 85 mm Hg. or current use of blood pressure–lowering medication) in N=3071 participants with depressed mood.**

|  | Odds Ratio | P | 95% Confidence Interval  Odds Ratio | |
| --- | --- | --- | --- | --- |
| Simple model with interactions: |  |  |  |  |
| *Child maltreatment - 1 type vs 0 types* | 0.773 | .144 | 0.547 | 1.092 |
| *2 vs 0* | 0.925 | .712 | 0.612 | 1.398 |
| *3 vs 0* | 0.742 | .111 | 0.514 | 1.071 |
| *4 vs 0* | 0.687 | .241 | 0.366 | 1.287 |
| *Sex - female vs male* | 0.474 | <.001 | 0.376 | 0.598 |
| *Sex * Child maltreatment – 1 type vs 0 types* | 1.179 | .466 | 0.757 | 1.836 |
| *Sex * Child maltreatment - 2 types vs 0 types* | 0.871 | .589 | 0.528 | 1.437 |
| *Sex* Child maltreatment - 3 types vs 0 types* | 1.262 | .330 | 0.790 | 2.017 |
| *Sex * Child maltreatment - 4 types vs 0 types* | 1.696 | .158 | 0.815 | 3.529 |
| *Constant* | 1.407 | <.001 | 1.163 | 1.702 |
| Simple model without interactions: |  |  |  |  |
| *Child maltreatment - 1 type vs 0 types* | 0.861 | .153 | 0.701 | 1.057 |
| *2 vs 0* | 0.850 | .188 | 0.666 | 1.084 |
| *3 vs 0* | 0.860 | .192 | 0.686 | 1.079 |
| *4 vs 0* | 1.022 | .891 | 0.751 | 1.391 |
| *Sex - female vs male* | 0.511 | <.001 | 0.439 | 0.595 |
| *Constant* | 1.337 | <.001 | 1.153 | 1.551 |
| Full model without: |  |  |  |  |
| *Child maltreatment - 1 type vs 0 types* | 0.912 | .438 | 0.722 | 1.152 |
| *2 vs 0* | 0.930 | .606 | 0.705 | 1.227 |
| *3 vs 0* | 0.741 | .026 | 0.569 | 0.965 |
| *4 vs 0* | 0.946 | .761 | 0.662 | 1.351 |
| *Constant* | 0.040 | <.001 | 0.022 | 0.072 |

Full model: adjusted for sex, age, educational level, smoking, alcohol use, psychopharmacological medications, ethnicity (reference group Dutch), any negative life events (12 months).

**Supplementary Table 6C. Results of the logistic regression analysis assessing the association between number of experienced child maltreatment types and metabolic syndrome component reduced HDL cholesterol (< 1.0 mM for men. < 1.3 mM for women. or current use of lipid-lowering medication) in N=3061 participants with depressed mood.**

|  | Odds Ratio | P | 95% Confidence Interval  Odds Ratio | |
| --- | --- | --- | --- | --- |
| Simple model with interactions: |  |  |  |  |
| *Child maltreatment - 1 type vs 0 types* | 1.217 | .276 | 0.855 | 1.733 |
| *2 vs 0* | 0.954 | .817 | 0.643 | 1.417 |
| *3 vs 0* | 0.854 | .422 | 0.581 | 1.255 |
| *4 vs 0* | 1.069 | .836 | 0.569 | 2.007 |
| *Sex - female vs male* | 1.168 | .191 | 0.925 | 1.475 |
| *Sex * Child maltreatment – 1 type vs 0 types* | 0.761 | .229 | 0.488 | 1.189 |
| *Sex * Child maltreatment - 2 types vs 0 types* | 0.870 | .566 | 0.540 | 1.401 |
| *Sex* Child maltreatment - 3 types vs 0 types* | 1.017 | .945 | 0.625 | 1.654 |
| *Sex * Child maltreatment - 4 types vs 0 types* | 0.890 | .752 | 0.433 | 1.830 |
| *Constant* | 0.664 | <.001 | 0.548 | 0.805 |
| Simple model without interactions: |  |  |  |  |
| *Child maltreatment - 1 type vs 0 types* | 1.017 | .878 | 0.819 | 1.263 |
| *2 vs 0* | 0.869 | .268 | 0.677 | 1.115 |
| *3 vs 0* | 0.860 | .215 | 0.678 | 1.092 |
| *4 vs 0* | 0.984 | .916 | 0.725 | 1.335 |
| *Sex - female vs male* | 1.080 | .329 | 0.926 | 1.259 |
| *Constant* | 0.701 | <.001 | 0.602 | 0.816 |
| Full model without: |  |  |  |  |
| *Child maltreatment - 1 type vs 0 types* | 1.060 | .618 | 0.843 | 1.332 |
| *2 vs 0* | 0.980 | .878 | 0.752 | 1.276 |
| *3 vs 0* | 0.887 | .345 | 0.692 | 1.138 |
| *4 vs 0* | 1.175 | .335 | 0.846 | 1.631 |
| *Constant* | 0.146 | <.001 | 0.085 | 0.250 |

Full model: adjusted for sex, age, educational level, smoking, alcohol use, psychopharmacological medications, ethnicity (reference group Dutch), any negative life events (12 months).

**Supplementary Table 6D. Results of the logistic regression analysis assessing the association between number of experienced child maltreatment types and metabolic syndrome component elevated triglycerides (≥ 1.7 mM. or current use of lipid-lowering medication) in N=3060 participants with depressed mood.**

|  | Odds Ratio | P | 95% Confidence Interval  Odds Ratio | |
| --- | --- | --- | --- | --- |
| Simple model with interactions: |  |  |  |  |
| *Child maltreatment - 1 type vs 0 types* | 1.217 | .276 | 0.855 | 1.733 |
| *2 vs 0* | 0.954 | .817 | 0.643 | 1.417 |
| *3 vs 0* | 0.854 | .422 | 0.581 | 1.255 |
| *4 vs 0* | 1.069 | .836 | 0.569 | 2.007 |
| *Sex - female vs male* | 1.168 | .191 | 0.925 | 1.475 |
| *Sex * Child maltreatment – 1 type vs 0 types* | 0.761 | .229 | 0.488 | 1.189 |
| *Sex * Child maltreatment - 2 types vs 0 types* | 0.870 | .566 | 0.540 | 1.401 |
| *Sex* Child maltreatment - 3 types vs 0 types* | 1.017 | .945 | 0.625 | 1.654 |
| *Sex * Child maltreatment - 4 types vs 0 types* | 0.890 | .752 | 0.433 | 1.830 |
| *Constant* | 0.664 | <.001 | 0.548 | 0.805 |
| Simple model without interactions: |  |  |  |  |
| *Child maltreatment - 1 type vs 0 types* | 1.017 | .878 | 0.819 | 1.263 |
| *2 vs 0* | 0.869 | .268 | 0.677 | 1.115 |
| *3 vs 0* | 0.860 | .215 | 0.678 | 1.092 |
| *4 vs 0* | 0.984 | .916 | 0.725 | 1.335 |
| *Sex - female vs male* | 1.080 | .329 | 0.926 | 1.259 |
| *Constant* | 0.701 | <.001 | 0.602 | 0.816 |
| Full model without: |  |  |  |  |
| *Child maltreatment - 1 type vs 0 types* | 1.060 | .618 | 0.843 | 1.332 |
| *2 vs 0* | 0.980 | .878 | 0.752 | 1.276 |
| *3 vs 0* | 0.887 | .345 | 0.692 | 1.138 |
| *4 vs 0* | 1.175 | .335 | 0.846 | 1.631 |
| *Constant* | 0.146 | <.001 | 0.085 | 0.250 |

Full model: adjusted for sex, age, educational level, smoking, alcohol use, psychopharmacological medications, ethnicity (reference group Dutch), any negative life events (12 months).

**Supplementary Table 6E. Results of the logistic regression analysis assessing the association between number of experienced child maltreatment types and metabolic syndrome component elevated waist circumference (ethnic specific cut-off values; for all women ≥ 80 cm. South-Asian men ≥ 90 cm and other men ≥ 94 cm) in N=3076 participants with depressed mood.**

|  | Odds Ratio | P | 95% Confidence Interval  Odds Ratio | |
| --- | --- | --- | --- | --- |
| Simple model with interactions: |  |  |  |  |
| *Child maltreatment - 1 type vs 0 types* | 0.828 | .275 | 0.590 | 1.162 |
| *2 vs 0* | 0.878 | .527 | 0.586 | 1.315 |
| *3 vs 0* | 0.768 | .182 | 0.521 | 1.132 |
| *4 vs 0* | 0.856 | .630 | 0.454 | 1.612 |
| *Sex - female vs male* | 2.442 | <.001 | 1.901 | 3.136 |
| *Sex * Child maltreatment – 1 type vs 0 types* | 0.890 | .606 | 0.573 | 1.384 |
| *Sex * Child maltreatment - 2 types vs 0 types* | 0.879 | .624 | 0.525 | 1.471 |
| *Sex* Child maltreatment - 3 types vs 0 types* | 1.143 | .610 | 0.684 | 1.913 |
| *Sex * Child maltreatment - 4 types vs 0 types* | 1.405 | .405 | 0.631 | 3.128 |
| *Constant* | 1.651 | <.001 | 1.363 | 2.000 |
| Simple model without interactions: |  |  |  |  |
| *Child maltreatment - 1 type vs 0 types* | 0.774 | .025 | 0.619 | 0.968 |
| *2 vs 0* | 0.813 | .106 | 0.633 | 1.045 |
| *3 vs 0* | 0.826 | .137 | 0.642 | 1.062 |
| *4 vs 0* | 1.076 | .692 | 0.748 | 1.548 |
| *Sex - female vs male* | 2.426 | <.001 | 2.059 | 2.858 |
| *Constant* | 1.657 | <.001 | 1.417 | 1.939 |
| Full model without: |  |  |  |  |
| *Child maltreatment - 1 type vs 0 types* | 0.825 | .132 | 0.642 | 1.060 |
| *2 vs 0* | 0.924 | .574 | 0.701 | 1.218 |
| *3 vs 0* | 0.789 | .094 | 0.598 | 1.041 |
| *4 vs 0* | 1.200 | .368 | 0.807 | 1.783 |
| *Constant* | 0.039 | <.001 | 0.021 | 0.071 |

Full model: adjusted for sex, age, educational level, smoking, alcohol use, psychopharmacological medications, ethnicity (reference group Dutch), any negative life events (12 months).

**Supplementary Table 7. Results of the simple model assessing the association between number of experienced child maltreatment types and circulating CRP including maltreatment x sex interaction effects in N=5879 participants.**

|  | B | SE | t | p |
| --- | --- | --- | --- | --- |
| *Child maltreatment - 1 type vs 0 types* | 0.012 | 0.017 | 0.751 | .453 |
| *2 vs 0* | 0.025 | 0.022 | 1.148 | .251 |
| *3 vs 0* | 0.003 | 0.024 | 0.111 | .912 |
| *4 vs 0* | 0.020 | 0.051 | 0.390 | .696 |
| *Sex - female vs male* | 0.095 | 0.009 | 10.313 | <.001 |
| *Sex * Child maltreatment – 1 type vs 0 types* | -0.044 | 0.021 | -2.065 | .039 |
| *Sex * Child maltreatment - 2 types vs 0 types* | -0.034 | 0.030 | -1.161 | .246 |
| *Sex* Child maltreatment - 3 types vs 0 types* | -0.012 | 0.031 | -0.398 | .691 |
| *Sex * Child maltreatment - 4 types vs 0 types* | 0.011 | 0.058 | 0.188 | .851 |
| *Constant* | 0.371 | 0.007 | 53.562 | <.001 |
